# Evaluating the contribution of weather variables to machine learning forecasts of visceral leishmaniasis in Brazil

**DOI:** 10.1088/2752-5309/ae1ce2

**Published:** 2025-11-21

**Authors:** Quinn H Adams, Chad W Milando, Kayoko Shioda, Guilherme L Werneck, Alexander Rodríguez, Davidson H Hamer, Gregory A Wellenius

**Affiliations:** 1 Center for Climate and Health, Boston University School of Public Health, Boston, MA, United States of America; 2 Department of Environmental, Boston University School of Public Health, Boston, MA, United States of America; 3 Department of Global Health, Boston University School of Public Health, Boston, MA, United States of America; 4 Center on Emerging Infectious Diseases, Boston University, Boston, MA, United States of America; 5 Department of Epidemiology, Rio de Janeiro State University, Rio de Janeiro, Brazil; 6 Computer Science and Engineering, University of Michigan, Ann Arbor, MI, United States of America; 7 Section of Infectious Diseases, Department of Medicine, Boston University Chobanian & Avedisian School of Medicine, Boston, MA, United States of America

**Keywords:** climate sensitive infectious diseases, neglected tropical diseases, machine learning forecasting, public health decision support, weather-based early warning system

## Abstract

Visceral leishmaniasis (VL), a deadly neglected tropical disease, remains a persistent public health challenge in Brazil, where transmission is shaped by interacting climatic, environmental, and sociodemographic factors. Despite evidence that weather conditions influence VL dynamics, they remain underutilized for outbreak prediction. This study evaluates whether climate-informed machine learning can support early warnings for VL in Brazil. We developed machine learning models to forecast monthly VL case counts and classify outbreak risk using data from 2007 to 2024 across 113 Brazilian municipalities. A cutting-edge sliding window approach enabled models to capture both short- and long-term trends using lagged meteorological data combined with land-use and sociodemographic variables. Risk classification models were developed for a subset of 22 municipalities following the Brazilian Ministry of Health’s prioritization framework to enable direct policy alignment. Predictive performance and variable importance were evaluated across locations. Weather patterns and indicators of human land-use pressure consistently ranked among the strongest predictors of VL risk. However, the relative importance of predictors varied across municipalities, reflecting local differences in transmission dynamics. Overall, forecasting models successfully captured long-term trends in observed case counts, and risk classification models, offering particularly timely and actionable signals for targeted intervention, achieved area under the curve scores above 0.80 in 86% of municipalities. Weather-informed machine learning models can provide timely, locally tailored predictions of VL risk in Brazil. As weather variability intensifies, integrating environmental data into existing surveillance systems may improve preparedness and reduce disease burden in vulnerable communities.

## 1. Introduction

Visceral leishmaniasis (VL) is a parasitic neglected tropical disease with a widespread impact across Brazil, disproportionately affecting vulnerable populations in resource-poor settings [1–3]. While the incidence of VL has steadily declined over the past decade, efforts to reduce the case fatality rates have been unsuccessful in recent years, intensifying the need to develop and implement new strategies to control transmission [4].

In Brazil, VL is both zoonotic and vector-borne, and transmission dynamics are shaped by complex interactions among climatic, socioeconomic, demographic, and ecological factors [1, 5]. VL is spread to humans through the bite of infected female phlebotomine sandflies, primarily *Lutzomyia longipalpis* in Brazil [6]. Climate-related variables, including temperature, humidity, and precipitation, play a role in directly shaping vector populations by influencing their development, survival, biting rates, and reproduction [7–10]. Socioeconomic determinants such as poverty, inadequate sanitation, and limited access to education exacerbate human exposure to VL and may hinder timely access to healthcare resources [3, 11]. Moreover, rapid urban expansion and land-use changes have intensified these risks, facilitating VL emergence in previously unaffected areas [5, 12]. Compounding these challenges, Brazil’s high prevalence of HIV/AIDS in certain regions increases susceptibility to symptomatic VL among immunocompromised individuals [13, 14]. The expanding geographic footprint and persistent burden of VL underscore the urgent need for proactive intervention.

In Brazil, VL is fatal in the majority of untreated cases, making timely intervention by Brazil’s VL Surveillance and Control Program (PVC-VL) essential. Current efforts focus on early diagnosis and treatment of human cases, alongside initiatives targeting vector control, management of canine reservoirs, and robust epidemiological surveillance [15]. However, the current strategies have been unsuccessful in preventing geographic expansion and a rise in VL-associated mortality. Additionally, the PVC-VL does not rely on climate-sensitive variables to identify priority areas for intervention. Instead, municipalities are classified into four risk tiers based on a long-term moving average of monthly VL cases: no transmission (zero cases), sporadic transmission (greater than zero but less than 2.4 cases), moderate transmission (2.4–4.4 cases), and intense transmission (greater than 4.4 cases). Municipalities classified as having moderate or intense transmission are targeted for continuous and ongoing interventions, including vector control and surveillance. In contrast, municipalities with sporadic transmission typically do not receive sustained or proactive interventions.

As a result, the effective management of climate-sensitive diseases like VL hinges on the timely identification of municipalities at risk of transitioning into higher transmission categories, particularly those moving from sporadic to moderate transmission, where ongoing intervention would be triggered but is not yet in place [16]. Leveraging robust and interpretable predictive models could enable the timely allocation of resources, targeted vector control, and community-level interventions before outbreaks escalate. This paradigm shift is critical for improving outcomes, particularly in municipalities where delayed treatment and response contribute to higher fatality rates [2, 17].

Like many vector-borne diseases, meteorological extremes play a critical role in VL transmission, with regionally distinct temporal effects on vector dynamics and disease risk. This variability underscores the need for spatially targeted and climate-adaptive public health strategies. Incorporating climate data and land-use metrics into predictive frameworks offers a promising opportunity to develop early warning systems at the local level that mitigate VL risks, particularly in urbanizing and ecologically sensitive areas. While past research efforts have employed traditional techniques such as seasonal autoregressive integrated moving average (SARIMA) models to predict VL incidence trends in Maranhão, Brazil, the application of machine learning approaches remains underexplored [18]. While SARIMA models incorporate seasonality, they are otherwise strictly autoregressive. Advanced machine learning models can leverage large, complex datasets to identify nonlinear relationships across diverse variables; these may be a transformative tool for VL forecasting. Machine learning approaches such as decision trees (random forest, XGBoost) and artificial neural networks (long-short term memory) have been successfully employed for other vector-borne diseases in diverse settings, underscoring their potential to address VL in Brazil amidst ongoing climate variability and land-use transformation [19–26]. If successful in the context of VL, machine-learned forecasting of VL risk would provide a foundation for managing VL, enabling more resilient public health responses in Brazil and comparable endemic regions.

In this study, we propose a novel comprehensive framework for forecasting VL incidence in Brazilian municipalities. By integrating diverse time-varying predictors (e.g. climate, urbanicity, and land use change) and spatial variables (e.g. socioeconomic status, altitude, and soil type), we employ advanced machine learning techniques to capture both temporal dependencies and spatial dynamics. Our approach offers a robust, data-driven foundation for the development of climate-based early warning systems. The findings have the potential to inform targeted, timely, and evidence-based public health responses to VL in Brazil and provide a scalable framework for addressing similar challenges among climate-sensitive diseases in other endemic regions.

## 2. Methods

### 2.1. Study area and data sources

Brazil offers a compelling setting for this research due to its diverse socio-environmental, demographic, and health characteristics. As the largest country in South America, Brazil encompasses a wide range of climates, from tropical rainforests to arid semi-deserts, offering a diverse landscape for studying environment and health interactions. The country is also experiencing substantial urbanization, with densely populated cities like São Paulo and Salvador exhibiting sharp socioeconomic inequalities, making it ideal for exploring the intersection of environmental exposures, social determinants of health, and health outcomes. Additionally, Brazil’s comprehensive public health system (SUS) collects robust surveillance and epidemiological data, providing high-quality datasets for modeling and analysis [27]. These factors position the country as a critical context for studying the intersection of climate, environmental change, and public health in diverse and dynamic settings.

In this study, we leveraged machine learning to predict monthly VL case counts across Brazilian municipalities using data from 2007 through 2024. We include a comprehensive range of spatially and temporally varying predictors, including weather characteristics, socioeconomic factors, and land-use data (table 1). Monthly confirmed VL cases were collected from the Brazilian Ministry of Health Information System for Notifiable Diseases (SINAN). Weather variables were sourced through the European Centre for Medium-Range Weather Forecasts’ fifth-generation reanalysis (ERA5) monthly averaged data [28]. Socioeconomic variables were obtained from the Brazilian Institute of Geography and Statistics (IBGE). The human footprint index, derived from eight measures of anthropogenic pressure on the environment—including the built environment, population density, electric infrastructure, cropland, pastureland, roads, railways, and navigable waterways—was calculated at the annual municipality level using publicly available datasets (table 1) [29, 30].

**Table 1.** Description of predictor variables used in the VL prediction models. Variables span climate, environmental, epidemiological, and sociodemographic domains. Climate and weather variables were included with lag structures to capture delayed effects on transmission. Lag 0 refers to the last observed month at forecast issue.

Category	Variable	Temporal resolution	Spatial resolution	Source
Climate & weather	Temperature (2 m, °C)	Monthly (lags 0–6)	9 km (gridded)	ERA5[28]
	Total Precipitation (mm)	Monthly (lags 0–6)	9 km (gridded)	ERA5
	Relative humidity (%)	Monthly (lags 0–6)	9 km (gridded)	ERA5
	Dew point temperature (°C)	Monthly (lags 0–6)	9 km (gridded)	ERA5
	Windspeed (from U/V components)	Monthly (lags 0–6)	9 km (gridded)	ERA5
	Saturation vapor pressure deficit	Monthly (lags 0–6)	9 km (gridded)	ERA5
	Specific humidity	Monthly (lags 0–6)	9 km (gridded)	ERA5
	Oceanic Niño Index (ONI)	Monthly (lags 0–6)	Niño 3.4 region	NOAA[31]

Environmental	Land Use Coverage (% Pasture, Forest, Crop, Urban)	Annual	Municipality	MapBiomas [32]
	Human footprint	Annual	Municipality	Williams *et al* & Keys *et al* [29,30]
	Altitude (m)	Static	Municipality	Alvares *et al* [33]
	Soil type	Static	9 km (gridded)	ERA5
	Köppen Climate Classification	Static	Municipality	Alvares *et al* [33]

Epidemiological	VL Case Count	Monthly (lags 1–12)	Municipality	SINAN [27]
	HIV Coinfection (%)	Monthly	Municipality	SINAN

Socio- demographic	% Urban Population	Monthly	Municipality	IBGE [34]
	% High School Education	2010	Municipality	IBGE
	% Unemployed	2010	Municipality	IBGE
	Average income among Employed	2010	Municipality	IBGE
	% of Households with electricity	2010	Municipality	IBGE
	Social vulnerability index (SVI)	2010	Municipality	IBGE
	Gini Index	2010	Municipality	IBGE
	Human development index (HDI)	2010	Municipality	IBGE

VL is a relatively rare disease, resulting in a high prevalence of zero-inflated case counts across many municipalities. To ensure robust model training, we focused on a subset of municipalities with sufficient case counts. Specifically, we selected municipalities reporting at least 10 cases every two years throughout the study period (2007–2024), resulting in a sample of 113 municipalities (figure S1). This approach minimized the influence of data sparsity while maintaining statistical power for model development [35].

### 2.2. Model development

Our study aimed to achieve three primary objectives: (a) using a regression-based forecasting model to predict VL case counts in advance across a broad set of municipalities, enabling targeted intervention planning; (b) developing a classification model to predict higher-risk transmission periods with sufficient lead time to support proactive public health responses; and (c) IDENTIFYING the most important predictors of VL risk to enhance interpretability and guide surveillance strategies.

To address these objectives, we used eXtreme Gradient Boosting (XGBoost) with a sliding window framework for both regression and classification modeling. First, we applied XGBoost regression to predict monthly VL case counts at a 3 month horizon for each municipality. Second, we implemented XGBoost classification in a subset of municipalities. We used the model’s 3 month ahead predictions to determine whether a municipality would be classified as moderate- or sporadic-risk based on Ministry of Health (MOH) intervention thresholds. This classification approach was designed to provide public health agencies with at least three months’ lead time to prepare for a potential increase in transmission risk and deploy control measures accordingly in areas on the verge of transitioning to higher risk levels. This model was used to assess which predictors were most important for VL risk prediction through SHapley Additive exPlanations (SHAP).

XGBoost is a supervised learning algorithm that trains models by minimizing prediction error through gradient descent while iteratively refining predictions by adding decision trees that correct errors made by previous trees. In time series analysis, data points are inherently dependent on one another due to temporal autocorrelation. XGBoost’s approach of sequentially updating predictions by correcting errors from previous trees makes it well-suited for capturing these temporal dependencies. Its ability to model nonlinear relationships and complex data structures further enhances its applicability. XGBoost has demonstrated superior performance in addressing complex, nonlinear time series problems compared to traditional methods such as ARIMA models [36, 37].

In addition to the primary 3 month ahead forecast, we also developed models to predict VL risk at 6- and 12 month horizons. These extended-horizon forecasts aim to assess the feasibility of longer lead times for intervention planning, given VL’s long incubation period and latent transmission cycles.

To capture potential lagged effects of climate on VL transmission, we developed lagged features for all weather-related variables, acknowledging that vector and parasite dynamics may respond to climate conditions with a delay. Similarly, lagged VL case counts were included to account for the autoregressive nature of VL transmission, where cases in one month are partially dependent on cases from prior months. To prevent target leakage, case counts enter as lags 1–12 (i.e. *t –* 1*…t −* 12), never contemporaneous with the target period. We incorporated a three-year moving average of VL cases, aligning with the MOHs current methodology for assessing transmission intensity in Brazilian municipalities [38]. We also constructed interaction terms between climate variables and pre-selected non-time-varying contextual features. All static covariates (e.g. SVI and HDI) were standardized using their global mean and standard deviation. Interaction terms were then defined as the multiplicative product of the standardized covariate and the corresponding climate variable (e.g. temp*SVI).

To ensure robustness in forecasting, we employed a sliding window framework that mimics real-world prediction scenarios[39–44]. At each iteration, models were trained on a 24 month historical window and validated on the subsequent 3 month period. This approach allowed models to adapt to seasonality, long-term trends, and environmental shifts while maintaining temporal separation between training and testing datasets. For example, in the first iteration, the model was trained on data from January 2007 to December 2008 and used to generate forecasts for January–March 2009. Because this was a retrospective exercise rather than an operational forecast, all lagged predictors were indexed relative to the target month using observed values. For example, for a forecast targeting January 2009, weather lag 0 corresponded to the observed January 2009 value, weather lag 1 to December 2008, and so on; similarly, case lag 1 corresponds to December 2008 and case lag 12 to January 2008. The 24 month historical window was selected to balance sufficient context, such as seasonality and other longer-term time trends, while remaining responsive to changes in the data.

Since each municipality may have different underlying transmission dynamics and data characteristics, we implemented Bayesian optimization to select the optimal model parameters for each location. This approach allowed us to tailor hyperparameters to the unique data structure of each municipality, improving predictive performance. Models were run separately for all municipalities to capture location-specific patterns. The methodological workflow is illustrated in figure S2.

### 2.3. Model evaluation

#### 2.3.1. Time series regression model

For each municipality, we applied a rolling three-month moving average as a smoothing technique to reduce noise in the monthly case data. To account for uncertainty in the regression-based predictions, we implemented a bootstrapping procedure, which involved repeatedly resampling from the training data with replacement (i.e. the same observation can be selected multiple times), fitting multiple models, and using the distribution of predictions to derive prediction intervals. For each of the 100 bootstrap datasets, an XGBoost model was trained, generating a distribution of predictions. Point estimates were calculated as the median prediction from these distributions, and uncertainty bounds were derived from the 2.5th and 97.5th percentiles. To evaluate model performance, coverage was calculated as the proportion of time points in which the observed value fell within the 95% prediction interval.

#### 2.3.2. Risk classification model

To put our model into context for public health and policy relevance for intervention deployment in Brazilian municipalities, we created a binary variable based on the three-year moving average case count for each municipality to identify the performance of an analogous XGBoost model, this time with binary classification as the main objective, in predicting when municipalities have ‘moderate’ or ‘sporadic’ transmission risk (table 2). Our definitions of transmission risk directly correspond to the definitions of risk used by the Brazilian MOH, which identifies areas of sporadic, moderate, and intense transmission [16]. Specifically, our classification distinguishes between sporadic transmission and moderate transmission because this is a transition point that triggers intervention based on the current MOH guidelines. Municipalities with sporadic transmission do not receive ongoing insecticide treatment, so the ability to predict an increase in risk class with enough lead time would allow for the implementation of interventions ahead of a rise in cases. This secondary analysis was limited to the 22 municipalities with at least one observed fluctuation between ‘sporadic’ and ‘moderate’ risk between 2007 and 2024. The models were run separately in each municipality (figure S1).

**Table 2.** Confusion matrix for binary classification of VL risk periods.

	Predicted (sporadic-risk)	Predicted (moderate-risk)
Observed (sporadic-risk)	True negative	False positive
Observed (moderate-risk)	False negative	True positive

To evaluate the predictive performance of the model, we computed the positive predictive value and sensitivity, also known as precision and recall, respectively, and the area under the curve (AUC) at each forecast horizon for each municipality.

\begin{equation*}{\text{Positive}}\;{\text{Predictive}}\;{\text{Value}}\left( {{\text{Precision}}} \right) = \frac{{{\text{True}}\;{\text{Positive}}}}{{{\text{ True}}\;{\text{Positive + False}}\;{\text{Positive}}}}.\end{equation*}

Interpretation: Probability that a month in a given municipality is classified as ‘moderate risk’ based on the observed moving average case counts, given that the model classifies that municipality as ‘moderate risk’.

\begin{equation*}{\text{Sensitivity }}\left( {{\text{Recall}}} \right) = {\text{ }}\frac{{{\text{True Positive}}}}{{{\text{True Positive}} + {\text{False Negative}}}}.\end{equation*}

Interpretation: Proportion of months in a given municipality considered ‘moderate risk’ that are correctly classified by the model as ‘moderate risk’.

We used SHAP to assess variable importance across 22 municipalities within 8 Brazilian states [45]. SHAP ranks feature importance by comparing model predictions with and without each feature across all possible feature combinations. This approach provides granular insights into the relative contributions of predictors, enabling a deeper understanding of the factors driving VL transmission and improving the model’s interpretability.

## 3. Results

### 3.1. Time series regression model

**Figure 1.**
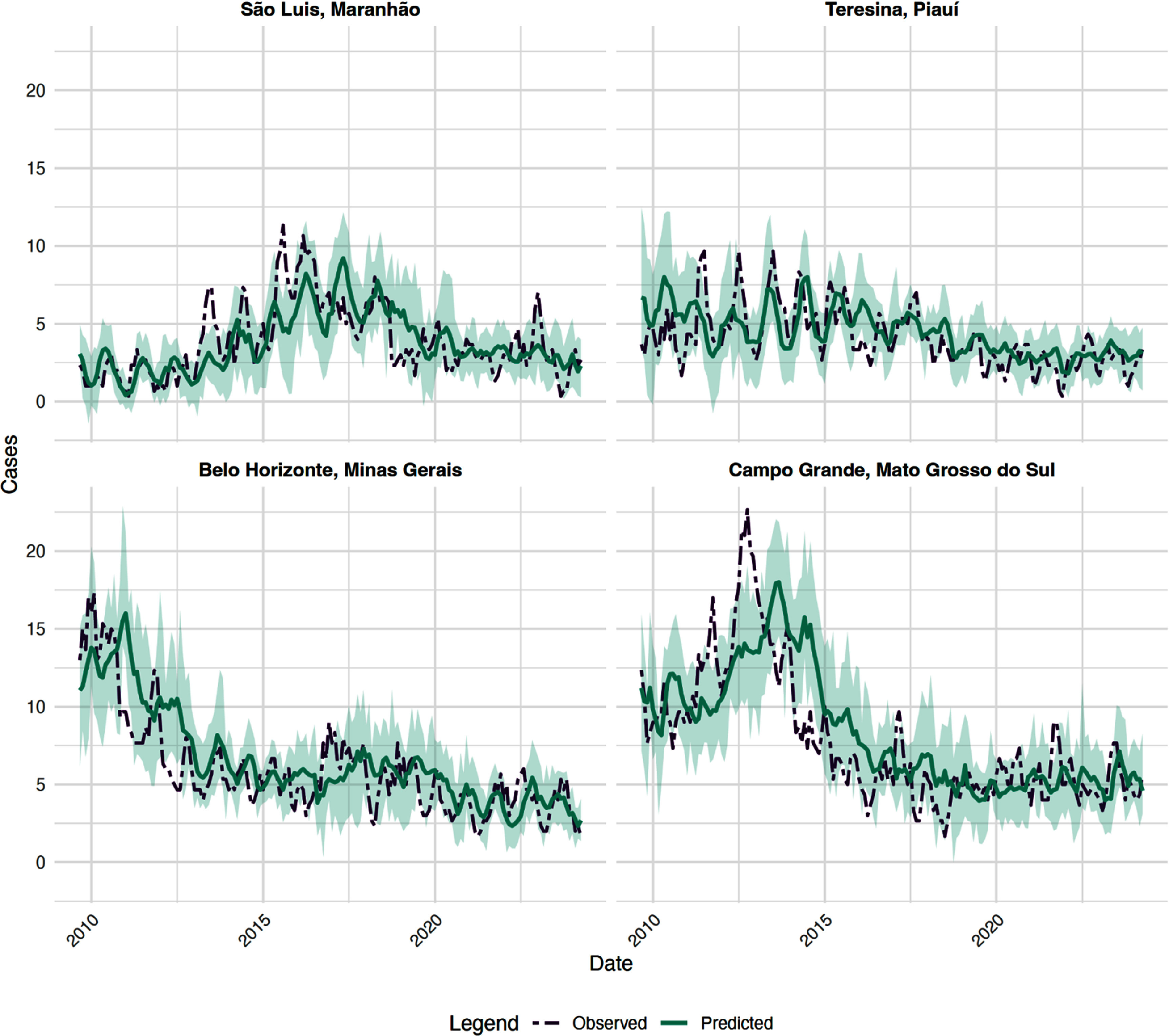
Time series of observed vs. predicted across municipalities with the highest coverage. Shaded teal areas represent the 95% prediction intervals for the predicted values.

The performance of the XGBoost case count forecast varied widely across municipalities. The coverage metric refers to the proportion of observed values that fall within the model’s 95% prediction interval, corresponding to a miscoverage rate (*α*) of 0.05. Coverage ranged from 50.0% to 88.1% with a median of 80.1%. We present the individual results in four municipalities located in different states, selected based on their high VL transmission intensity and priority for control measures. Predictions captured the average of observed trends in VL cases well across these municipalities (figure 1). Coverage for these municipalities was 81.3%, 81.8%, 77.8%, and 71.0% for São Luis, Teresina, Belo Horizonte, and Campo Grande, respectively.

### 3.2. Risk classification model

**Figure 2.**
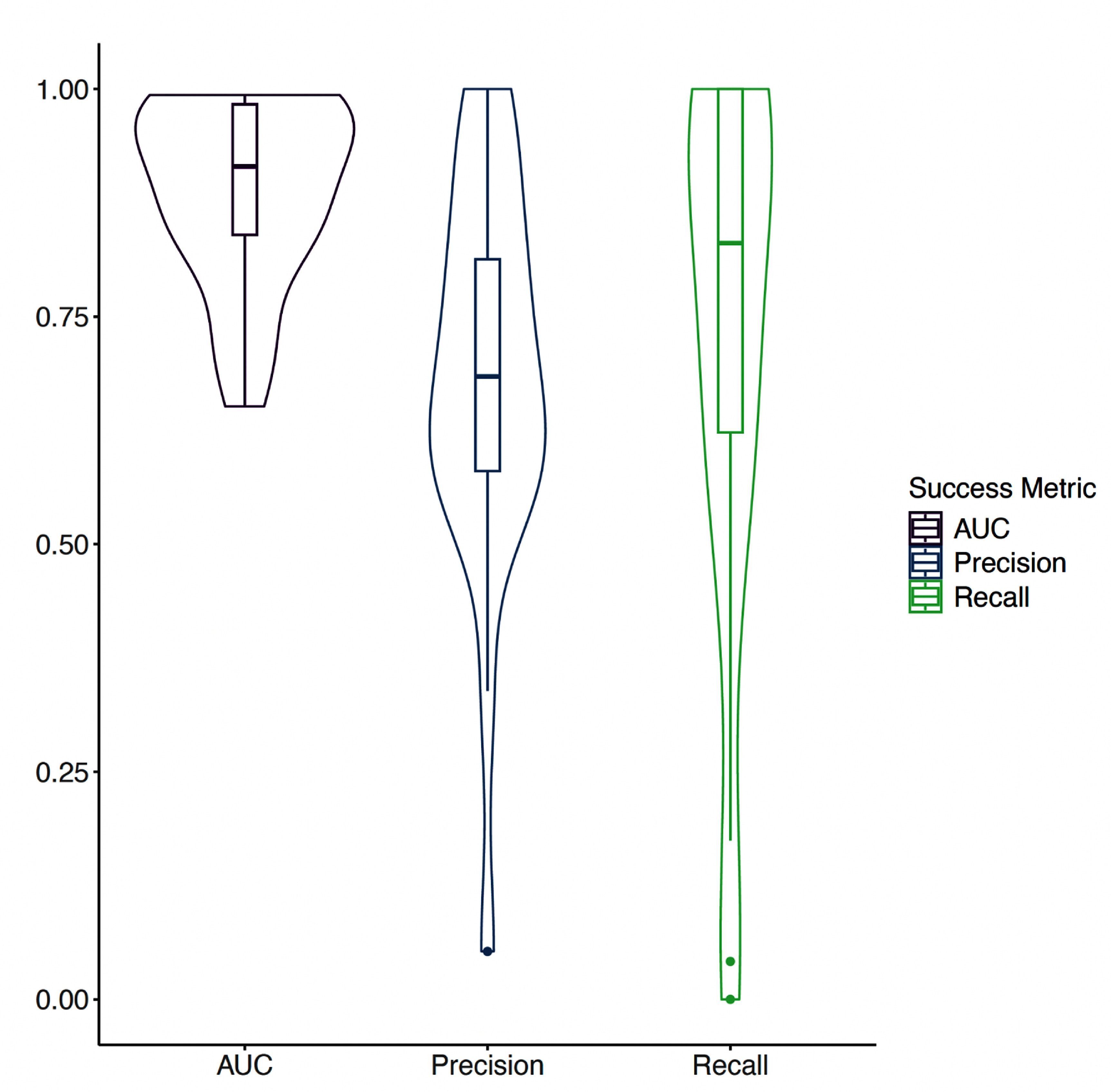
Distribution of the success metrics used to evaluate the classification model across 22 municipalities, showing variability in model discrimination (AUC), precision, and recall across locations. Boxplots inside of violin plots represent the median and interquartile range.

In our risk classification analysis of the 22 municipalities where VL cases fluctuated between moderate- and sporadic-risk categories based on MOH definitions, the model demonstrated strong predictive performance. AUC exceeded 0.8 in 18 out of 22 municipalities, indicating high discriminatory power in distinguishing between moderate- and sporadic-risk periods (figure 2). However, a subset of municipalities had markedly lower AUCs (AUC < 0.7). We observed wide variability across municipalities in how many false alarms were triggered (precision) and how many true positives were captured (recall). Precision exhibited greater variability than AUC, ranging from near-zero to above 0.9, with a central tendency around 0.75. This spread reflects differences in the model’s false positive rate across municipalities, likely influenced by class imbalance and underlying transmission patterns. Recall showed the widest range of all three metrics: in some municipalities, recall approached 1.0, indicating nearly all true positive periods were detected, while in others, it dropped to near-zero, suggesting the model failed to identify most moderate-risk periods. This variability in recall highlights the challenge of detecting rare or highly localized outbreaks, particularly in municipalities with sparse or noisy data.

In addition to the primary 3 month ahead forecasts, we evaluated model performance at 6- and 12 month horizons to assess the feasibility of earlier intervention planning. As expected, predictive performance declined modestly with increasing lead time. The median AUC for the 3 month forecast was 0.91 (range: 0.57–0.99), compared to 0.86 (range: 0.48–0.99) for the 6 month forecast and 0.84 (range: 0.52–0.98) for the 12 month forecast. Despite the decline, performance remained strong in many municipalities even at extended horizons, indicating that climate-informed models can detect early signals of elevated risk several months in advance.

**Figure 3.**
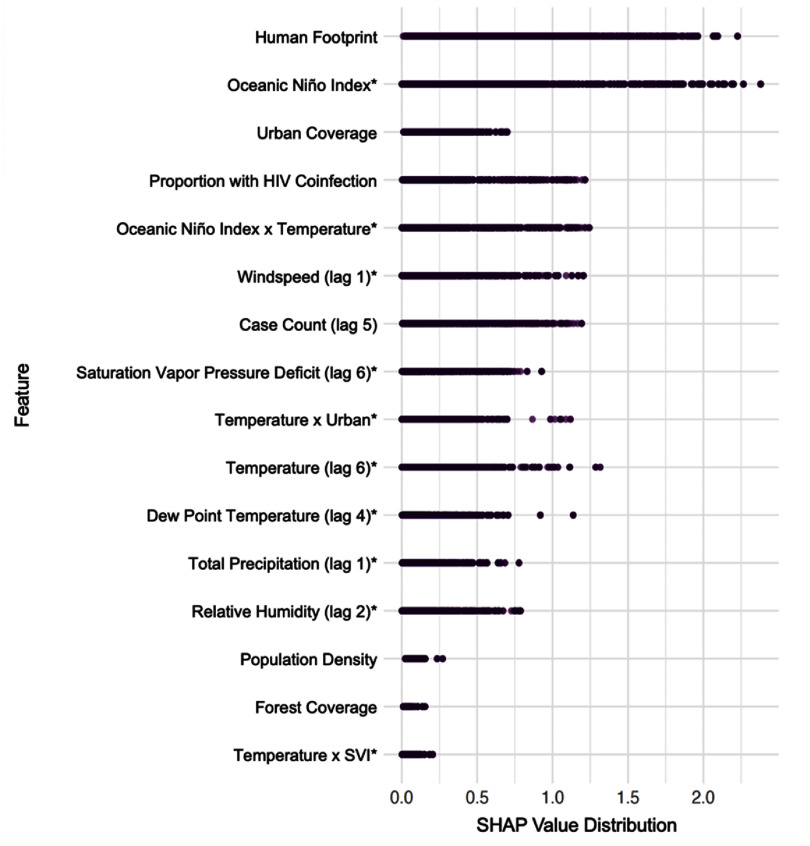
Distribution of the absolute value of mean SHAP values for a selected subset of predictors, illustrating variable importance across 22 municipalities. Features were chosen to represent the most important variable from each major thematic category (e.g. climate, land use, lagged incidence, sociodemographic factors) to avoid redundancy among lagged predictors. Variables labeled with an asterisk denote climate- or weather-related predictors. Interaction terms are products of standardized variables (constructed within municipality and month/lag).

To identify and rank the key drivers of VL risk, we quantified the feature importance using SHAP. Overall, many of the most important variables across the 22 municipalities were time-varying and directly linked to climate or weather (figure 3). The Oceanic Niño Index (ONI) was the most consistently important predictor of VL risk across municipalities and time windows. Multiple lags of ONI (0–6 months) ranked among the top predictors, indicating both immediate and delayed influences of large-scale climatic variability on disease dynamics. Human footprint—a composite indicator of anthropogenic activity and infrastructure—was the strongest non-climatic predictor, suggesting that land use and human exposure pathways play a central role in shaping transmission risk. Several lagged case count variables were also highly influential, consistent with known temporal autocorrelation and endemic persistence of VL. In addition, climate-related features such as wind speed, vapor pressure deficit, relative humidity, and dew point temperature emerged as important predictors, often with multi-month lags, reflecting the model’s sensitivity to delayed ecological effects on vector survival and transmission potential.

**Figure 4.**
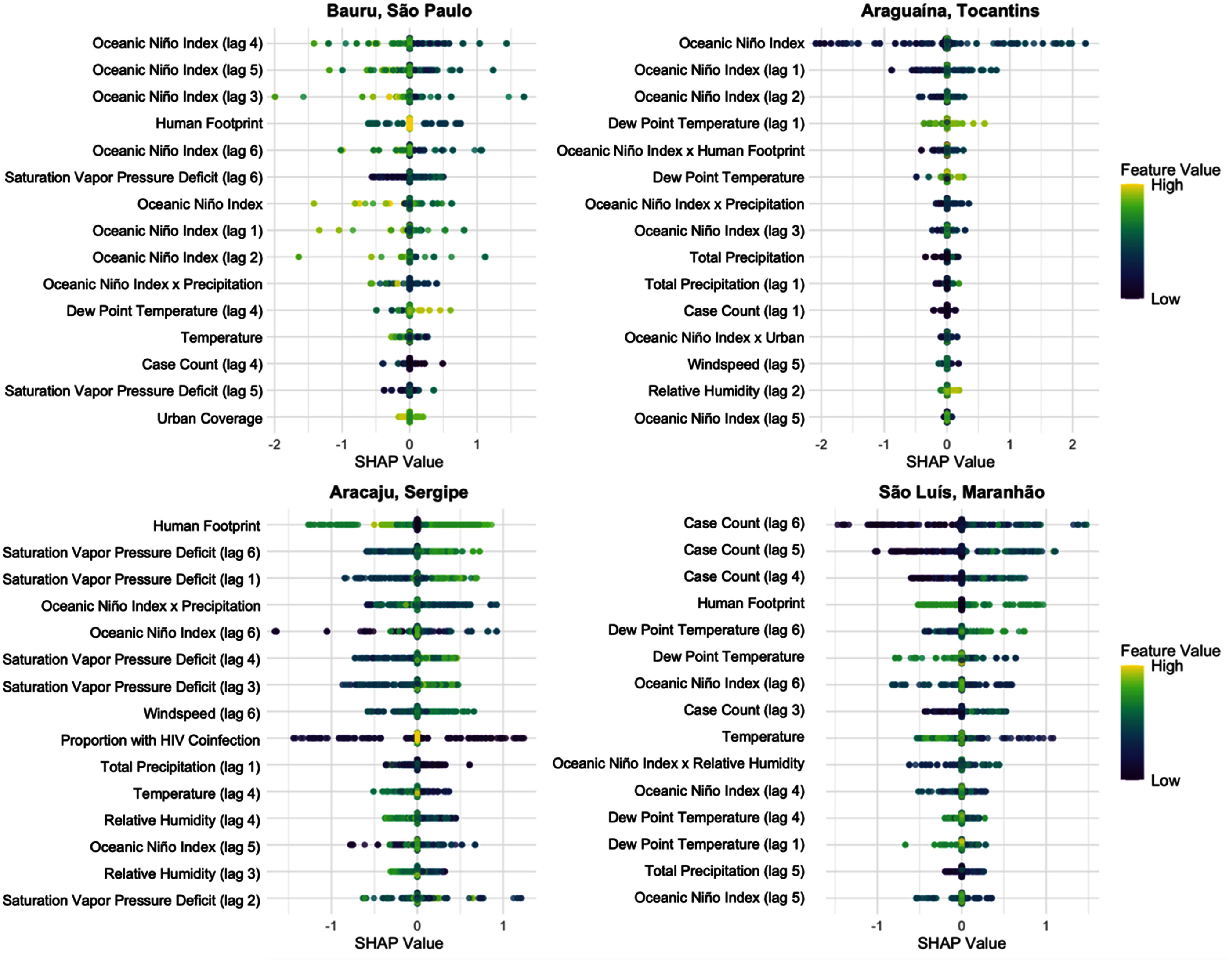
SHAP beeswarm plots showing the top 15 predictors of VL risk across four representative municipalities. Each point represents the SHAP value (i.e. the contribution of a feature to the model prediction) for a given time window, with point color indicating the corresponding feature value (low to high). Color gradients are scaled separately for each feature to account for differences in value ranges.

SHAP beeswarm plots for four representative municipalities revealed clear spatial variation in the drivers of predicted VL risk, with distinct patterns emerging in each location (figure 4). In these plots, the SHAP value represents the direction and magnitude of each feature’s contribution to the model’s prediction, while the color indicates the underlying feature value, allowing for simultaneous interpretation of both feature impact and intensity. For example, in Bauru (São Paulo), ONI-related variables dominated the feature set, with higher values of ONI, which are indicative of El Niño conditions, more commonly associated with negative SHAP values.

In contrast, São Luís (Maranhão) showed strong and consistent influence from lagged case counts, highlighting the influence of endemic persistence and local transmission memory in shaping VL risk. Araguaína (Tocantins) was more sensitive to a mix of ONI, dew point temperature, precipitation, and humidity, suggesting that local meteorological fluctuations play a greater role in shaping risk there. Aracaju (Sergipe) showed a more evenly distributed mix of predictors, including ONI, windspeed, and land use variables such as human footprint, indicating a broader integration of climate, environment, and structural factors.

Across all locations, ONI appeared frequently but with varying magnitude and direction; some high ONI values were associated with increased risk, others with decreased risk. These differences across geographies highlight the potentially nonlinear and context-dependent influence of ONI. This variability was mirrored in other features as well: for example, high values of precipitation or ONI could lead to both increased and decreased risk, depending on co-occurring conditions. Human footprint also remained consistently influential, though its exact contribution varied across municipalities. Overall, these results emphasize that the model is capable of capturing local epidemiological, climatic, and land-use contexts, and they highlight the importance of considering location-specific drivers in spatially resolved disease prediction models.

Corresponding SHAP dependence plots for each predictor and municipality (figure 4) are provided in supplementary figures S2–5 to illustrate full predictor-res

### 3.3. Classification and regression model performance comparison

To compare model performance between our time series case count prediction and risk classification forecast models, we calculated the Spearman correlation coefficient comparing regression and risk classification model performance across the 22 municipalities evaluated in both models. We found that there is no consistent relationship across locations (*ρ* = 0.25, *p* = 0.27). In other words, neither model outperformed the other consistently across all locations.

To further evaluate this result, we examined the relative differences between accuracy, calculated as the normalized AUC (classification model) minus the normalized percent coverage within the 95% prediction interval (case count forecasting model). To enable direct comparison between these metrics, we applied min-max normalization to both AUC and coverage values, rescaling them to a common 0–1 scale. This approach accounts for differences in scale and facilitates location-specific comparisons. The resulting performance gap varied substantially across locations (figure S7), highlighting heterogeneity in model performance and suggesting that the relative difficulty of classification versus regression tasks is context dependent.

## 4. Discussion

Understanding how climatic, environmental, and sociodemographic factors interact to shape vector-borne disease dynamics remains a fundamental challenge, due to their nonlinear, lagged, and interacting effects. Traditional time-series models often fall short in capturing these complexities. In this study, we used machine learning to forecast VL using a flexible modeling framework capable of identifying complex, location-specific drivers of risk. Accurate predictions made several months in advance could shift public health strategies from reactive case management toward proactive, preventive interventions.

Our results provide compelling evidence for the critical role of climatic and weather-related factors in shaping VL dynamics across diverse Brazilian municipalities. Weather variables, including temperature, precipitation, wind speed, and humidity, consistently ranked among the top predictors of VL incidence. Temperature-related predictors, including lagged surface temperature and derived metrics such as saturation vapor pressure deficit and dew point temperature, also consistently ranked among the most influential features in the model. While the relative importance of these variables varied by location and time, their recurrent appearance across municipalities highlights the sustained influence of thermal and moisture conditions in modulating transmission dynamics. These findings are supported by entomological studies, which demonstrate that the survival and development of *Lutzomyia longipalpis*, the primary vector of VL in Brazil, are highly temperature-sensitive [8]. However, the biology of sandflies is complex: while warmer temperatures may increase transmission efficiency, cooler temperatures can extend adult sandfly survival, particularly among females [7, 46]. This dual influence suggests that both short-term temperature anomalies and seasonal thermal trends can have significant, and sometimes counterbalancing, effects on VL transmission. The inclusion of lagged temperature features and interaction terms (e.g. temperature × urbanicity) further supports the notion that climatic impacts on transmission are not isolated but instead shaped by underlying environmental and social contexts.

The ONI emerged as a particularly important driver, aligning with major El Niño years and highlighting the broader influence of large-scale climatic variability. Human land use, captured via the human footprint index, was also persistently influential, emphasizing the role of urban expansion and environmental change in VL emergence. These findings suggest that VL risk is shaped not only by short-term climatic fluctuations but also by long-term landscape changes driven by human activity [47].

While ONI (lag 0) emerged as a dominant climate predictor in Araguaína, we caution against interpreting this as a universal of mechanistically direct ENSO effect. ENSO teleconnections in Northern Brazil are known to operate through region- and season-specific rainfall and soil moisture anomalies, with some locations exhibiting near-immediate responses while other experience more delayed hydrological impacts [48]. The prominence of ONI in Araguaína may reflect contemporaneous anomalies in boreal winter-spring or upstream hydro-climatic information that are relevant for short lags as in our framing [49, 50]. However, as shown in figure 4, the direction of the effect is context-specific and is likely mediated by local rainfall and moisture pathways among other co-occurring factors.

Importantly, the relative importance of predictors varied substantially by location. Some municipalities were more sensitive to climatic fluctuations, while others were more affected by persistent structural factors. This spatial heterogeneity reinforces the value of flexible, subnational forecasting tools that can be tuned to local transmission dynamics. Our use of SHAP values enabled interpretable, municipality-specific insights into model behavior.

While the regression-based model successfully captured the smoothed trend in case counts using a 3 month moving average, it struggled to reproduce high-frequency variability in monthly data. To address this limitation, we implemented a classification model using the same predictors to identify transitions into elevated-risk periods, defined by a MOH intervention threshold of 2.4 cases. The classification model performed well overall, achieving AUC values above 0.80 in 18 of 22 municipalities, with several achieving high sensitivity and minimal false positives. This indicates the model’s ability to detect subtle shifts in transmission dynamics before they escalate. However, performance was lower in municipalities with few or infrequent transitions, underscoring the challenge of predicting rare events in low-incidence settings. Future improvements could involve integrating real-time entomological surveillance data, such as sandfly abundance and canine infection prevalence, to enhance model accuracy in regions where risk classification remains uncertain.

While predictive performance was highest for 3 month ahead forecasts, we also found that meaningful signals could be detected up to 6–12 months in advance. These findings have important implications for early warning system design. Short-term forecasts may be particularly valuable for triggering enhanced surveillance and case detection efforts to ensure timely treatment. In contrast, longer-horizon forecasts, despite slightly reduced accuracy, may be better suited for initiating preventive measures such as insecticide spraying or reservoir control, which aim to interrupt transmission before human infection occurs. This distinction is especially relevant for VL, where the incubation period can span several months, meaning that individuals infected today may not present clinically until well after the opportunity for upstream intervention has passed.

Classification and regression models both offer valuable but differing insights for early warning systems. Our performance gap analysis revealed that their relative strengths vary substantially across municipalities. Early warning systems may benefit from hybrid modeling approaches in which classification models are used to flag periods of elevated risk requiring immediate attention, while regression models support longer-term trend monitoring and resource allocation. Ultimately, incorporating model performance diagnostics into operational workflows for early warning systems enhances the robustness and credibility of early warning efforts in diverse settings.

Despite VL’s well-established association with poverty and structural vulnerability, sociodemographic variables such as the social vulnerability index (SVI) and average income had low relative importance in the classification model. This was somewhat surprising given VL’s historical link to socioeconomic deprivation [1, 2, 51, 52]. One likely explanation is the limited availability of temporally resolved SES data. Most indicators, such as the Human Development Index, SVI, and education data are only updated during census years and may not reflect recent demographic or infrastructural shifts at the municipal level. As a result, their utility in short-term risk classification is limited. Additionally, the weak influence of socioeconomic variables may suggest that climatic and ecological signals dominate near-term transmission patterns, whereas structural factors may operate more strongly at longer time scales or shape baseline vulnerability rather than temporal risk.

This study has several limitations. First, forecasting VL remains inherently difficult due to complex, nonlinear interactions between environmental and biological processes. Our data-driven framework does not explicitly capture vector or reservoir biology, as we lacked entomological data such as sandfly abundance and canine infection prevalence, critical components for understanding local transmission dynamics. Integrating mechanistic or hybrid models could strengthen causal inference and improve the interpretation of intervention effects.

Second, this analysis used a retrospective framework that depends on historical weather and case data. Real-time applications would require access to reliable climate forecasts and up-to-date case data, resources not currently available across all regions. Third, the rarity of VL in many municipalities introduces challenges in model training and evaluation, especially when case counts are too low to distinguish signal from noise. To mitigate overfitting, our main classification analysis was restricted to 22 municipalities with known historical transitions between risk categories. This selection, informed by retrospective knowledge of past risk transitions, may overestimate real-world performance. Finally, although our municipality-specific models were designed to capture local heterogeneity and improve forecast performance, this approach does not incorporate cross-location information among neighboring municipalities. Future research should evaluate hierarchical or pooled strategies in the context of VL forecasting to better balance local specificity with shared structures across municipalities.

Despite these challenges, our results support a growing body of evidence that climate-informed machine learning models can provide scalable, flexible tools for disease forecasting [19–22, 26, 37, 40]. These models are particularly valuable in resource-limited settings, where traditional epidemiological surveillance may be fragmented or delayed.

## 5. Conclusion

Our findings represent a meaningful step toward the development of climate-based early warning systems for VL and other climate-sensitive vector-borne diseases. This study demonstrates that weather conditions and land use patterns are key drivers of VL dynamics, influencing transmission across diverse environmental and sociodemographic contexts. By applying machine learning techniques, we capture complex, nonlinear interactions and shifting patterns of influence over time, capabilities that exceed those of traditional models. Aligned with national intervention thresholds, our classification model provides interpretable, policy-relevant insights to support earlier and more targeted responses. As climate variability accelerates and public health systems face mounting challenges, predictive tools offer a scalable and evidence-based approach to reducing transmission risk and improving resource allocation in vulnerable settings.

## Data Availability

The data that support the findings of this study are openly available at the following URL/DOI: https://github.com/qadams13/ml-vl-forecasting. Supplementary data available at https://doi.org/10.1088/2752-5309/ae1ce2/data1.

## References

[1] Valero N N H, Prist P, Uriarte M (2021). Environmental and socioeconomic risk factors for visceral and cutaneous leishmaniasis in São Paulo, Brazil. Sci. Total Environ..

[2] Cota G, Erber A C, Schernhammer E, Simões T C, Ramos A N (2021). Inequalities of visceral leishmaniasis case-fatality in Brazil: a multilevel modeling considering space, time, individual and contextual factors. PLoS Negl. Trop. Dis..

[3] Werneck G L, Braga E (2019). Socioeconomic factors predict the increase of incidence rates of visceral leishmaniasis in higly endemic areas in Brazil. Int. J. Infect. Dis..

[4] Bruhn F R P (2024). Spatio-temporal dynamics of visceral leishmaniasis in Brazil: a nonlinear regression analysis. Zoonoses Public Health.

[5] Soares Santana R (2021). Cases and distribution of visceral leishmaniasis in western São Paulo: a neglected disease in this region of Brazil. PLoS Negl. Trop. Dis..

[6] Rocha M F (2022). Impact of vector control actions in the abundance of Lutzomyia longipalpis in Montes Claros, Brazil. Acta Trop..

[7] Guzmán H, Tesh B (2000). Effects of temperature and diet on the growth and longevity of phlebotomine sand flies. Biomédica.

[8] Hlavacova J, Votypka J, Volf P (2013). The effect of temperature on Leishmania (Kinetoplastida: trypanosomatidae) development in sand flies. J. Med. Entomol..

[9] Ximenes M, Castellón E G, de Souza M D F, Menezes A A L, Queiroz J W, Silva V P M E, Jerônimo S M B (2006). Effect of abiotic factors on seasonal population dynamics of Lutzomyia longipalpis (Diptera: psychodidae) in northeastern Brazil. J. Med. Entomol..

[10] Duque-Granda D, Vivero-Gómez R J, Junca H, Cadavid-Restrepo G, Moreno-Herrera C X (2024). Interaction and effects of temperature preference under a controlled environment on the diversity and abundance of the microbiome in Lutzomyia longipalpis (Diptera: psychodidae). Biotechnol. Rep..

[11] Vasconcelos A, Bedoya-Pacheco S J, Cunha E Silva R R, Magalhães M D A F M, de Sá T P S O, Dias C M G, Meneguete P S, de Almeida P M P, Pimentel M I F (2024). Spatial-temporal distribution of visceral leishmaniasis in Rio de Janeiro, Brazil, 2001–2020: expansion and challenges. Trans. R. Soc. Trop. Med. Hyg..

[12] Anversa L, Tiburcio M G S, Richini-Pereira V B, Ramirez L E (2018). Human leishmaniasis in Brazil: a general review. Rev. Assoc. Med. Bras..

[13] Belo V S, Struchiner C J, Barbosa D S, Nascimento B W L, Horta M A P, da Silva E S, Werneck G L (2014). Risk factors for adverse prognosis and death in American visceral leishmaniasis: a meta-analysis. PLoS Negl. Trop. Dis..

[14] Alvar J, Aparicio P, Aseffa A, Den Boer M, Cañavate C, Dedet J-P, Gradoni L, Ter Horst R, Loépez-Veélez R, Moreno J (2008). The relationship between leishmaniasis and AIDS: the second 10 years. Clin. Microbiol. Rev..

[15] França-Silva J C, Giunchetti R C, Mariano R M D S, Machado-Coelho G L L, Teixeira L D A S, Barata R A, Michalsky É M, Rocha M F, Fortes-Dias C L, Dias E S (2023). The Program for the control of Visceral Leishmaniasis in Brazil: the effect of the systematic euthanasia of seropositive dogs as a single control action in porteirinha, a Brazilian city with an intense transmission of Visceral Leishmaniasis. Pathogens.

[16] da Saúde www. saude. gov. br/bvs B. V. em S. do M. MINISTÉRIO DA SAÚDE. https://bvsms.saude.gov.br/bvs/publicacoes/manual_vigilancia_controle_leishmaniose_visceral_1edicao.pdf.

[17] de Carvalho I P S F, Peixoto H M, Romero G A S, de Oliveira M R F (2019). Treatment for human visceral leishmaniasis: a cost-effectiveness analysis for Brazil. Trop. Med. Int. Health.

[18] Pimentel K B A, Oliveira R S, Aragão C F, Aquino Júnior J, Moura M E S, Guimarães-e-silva A S, Pinheiro V C S, Gonçalves E G R, Silva A R (2022). Prediction of visceral leishmaniasis incidence using the Seasonal Autoregressive Integrated Moving Average model (SARIMA) in the state of Maranhão, Brazil. Braz. J. Biol..

[19] Farooq Z, Rocklöv J, Wallin J, Abiri N, Sewe M O, Sjödin H, Semenza J C (2022). Artificial intelligence to predict West Nile virus outbreaks with eco-climatic drivers. Lancet Reg. Health Eur..

[20] Meng D, Xu J, Zhao J, Singh R (2021). Analysis and prediction of hand, foot and mouth disease incidence in China using Random Forest and XGBoost. PLoS One.

[21] Donizette A C, Rocco C D, de Queiroz T A (2024). Predicting leishmaniasis outbreaks in Brazil using machine learning models based on disease surveillance and meteorological data. Oper. Res. Health Care.

[22] Mulwa D, Kazuzuru B, Misinzo G, Bett B (2024). An XGBoost approach to predictive modelling of Rift Valley fever outbreaks in Kenya using climatic factors. Big Data Cogn. Comput..

[23] Sun H, Chen S, Li X, Cheng L, Luo Y, Xie L (2023). Prediction and early warning model of mixed exposure to air pollution and meteorological factors on death of respiratory diseases based on machine learning. Environ. Sci. Pollut. Res. Int..

[24] Aleixo R, Kon F, Rocha R, Camargo M S, De Camargo R Y (2022). Predicting dengue outbreaks with explainable machine learning.

[25] Tuan D A, Dang T N (2024). Leveraging climate data for dengue forecasting in Ba Ria Vung Tau province, Vietnam: an advanced machine learning approach. Trop. Med. Infect. Dis..

[26] Sebastianelli A (2024). A reproducible ensemble machine learning approach to forecast dengue outbreaks. Sci. Rep..

[27] Ministério da Saúde Brasil SINAN Brasil Leishmaniose Visceral - Casos confirmados notificados no Sistema de Informação de Agravos de Notificação.

[28] Hersbach H (2020). The ERA5 global reanalysis. Q. J. R. Meteorol. Soc..

[29] Williams B A (2020). Change in terrestrial human footprint drives continued loss of intact ecosystems. One Earth.

[30] Keys P W, Barnes E A, Carter N H (2021). A machine-learning approach to human footprint index estimation with applications to sustainable development. Environ. Res. Lett..

[31] NOAA’s Climate Prediction Center (2001). NOAA’s Climate Prediction Center.

[32] Souza C M (2020). Reconstructing three decades of land use and land cover changes in Brazilian biomes with Landsat archive and earth engine. Remote Sens..

[33] Alvares C A, Stape J L, Sentelhas P C, de Moraes Gonçalves J L, Sparovek G (2013). Köppen’s climate classification map for Brazil. Meteorol Z..

[34] Instituto Brasileiro de Geografia e Estatística (IBGE) Censo Demográfico. www.ibge.gov.br/en/home-eng.html.

[35] Adams Q Code and data for: Evaluating the contribution of weather variables to machine learning forecasts of visceral leishmaniasis in Brazil. https://github.com/qadams13/ml-vl-forecasting.

[36] Lv C-X, An S-Y, Qiao B-J, Wu W (2021). Time series analysis of hemorrhagic fever with renal syndrome in mainland China by using an XGBoost forecasting model. BMC Infect. Dis..

[37] Dong B (2022). Spatio-temporal dynamics of three diseases caused by Aedes-borne arboviruses in Mexico. Commun. Med..

[38] Machado G, Alvarez J, Bakka H C, Perez A, Donato L E, de Ferreira Lima Júnior F E, Alves R V, Del Rio Vilas V J (2019). Revisiting area risk classification of visceral leishmaniasis in Brazil. BMC Infect. Dis..

[39] Ferdousi T, Cohnstaedt L W, Scoglio C M (2021). A windowed correlation-based feature selection method to improve time series prediction of dengue fever cases. IEEE Access.

[40] Schneider R (2021). Climate-based ensemble machine learning model to forecast dengue epidemics.

[41] Chen C, He Z, Zhao J, Zhu X, Li J, Wu X, Chen Z, Chen H, Jia G (2024). Zoonotic outbreak risk prediction with long short-term memory models: a case study with schistosomiasis, echinococcosis, and leptospirosis. BMC Infect. Dis..

[42] Rajendran N M (2023). Communicable disease prediction using machine learning and deep learning algorithms. Lecture Notes in Networks and Systems.

[43] Kim J, Lawson A B, Neelon B, Korte J E, Eberth J M, Chowell G (2023). Evaluation of Bayesian spatiotemporal infectious disease models for prospective surveillance analysis. BMC Med. Res. Methodol..

[44] Ratnavale S, Hepp C, Doerry E, Mihaljevic J R (2022). A sliding window approach to optimize the time-varying parameters of a spatially-explicit and stochastic model of COVID-19. PLOS Glob. Public Health.

[45] Lundberg S, Lee S-I, von Luxburg U, Guyon I (2017). A unified approach to interpreting model predictions.

[46] Martins K A (2022). Response to thermal and infection stresses in an American vector of visceral leishmaniasis. Med. Vet. Entomol..

[47] Skinner E B, Glidden C K, MacDonald A J, Mordecai E A (2023). Human footprint is associated with shifts in the assemblages of major vector-borne diseases. Nat. Sustain..

[48] Foley J A, Botta A, Coe M T, Costa M H (2002). El Niño–Southern oscillation and the climate, ecosystems and rivers of Amazonia: IMPACT OF ENSO ON AMAZONIAN ECOSYSTEMS AND RIVERS. Glob. Biogeochem. Cycles.

[49] Franke C R, Ziller M, Staubach C, Latif M (2002). Impact of the El Niño/Southern Oscillation on visceral leishmaniasis, Brazil. Emerg. Infect. Dis..

[50] Rodrigues R R, Haarsma R J, Campos E J D, Ambrizzi T (2011). The impacts of inter–El Niño variability on the tropical Atlantic and northeast Brazil climate. J. Clim..

[51] Andrade A W F, Souza C D F, Carmo R F (2020). Analysis of spatial clustering, time trend, social vulnerability and risk of human visceral leishmaniasis in an endemic area in Brazil: an ecological study. Trans. R. Soc. Trop. Med. Hyg..

[52] Nunes B E B R, Leal T C, Paiva J P S D, Silva L F D, Carmo R F D, Machado M F, Araújo M D P D, Santos V S, Souza C D F D (2019). Social determinants of mortality due to visceral leishmaniasis in Brazil (2001–2015): an ecological study. Rev. Soc. Bras. Med. Trop..

